# Supplementary material for: Structural basis for SARS-CoV-2 envelope protein recognition of human cell junction protein PALS1
Source: Nat Commun. 2021 Jun 8;12:3433. doi: 10.1038/s41467-021-23533-x (PMC8187709; doi:10.1038/s41467-021-23533-x)
Supplement: Supplementary file 1 — Supplementary Information [file 41467_2021_23533_MOESM1_ESM.pdf]

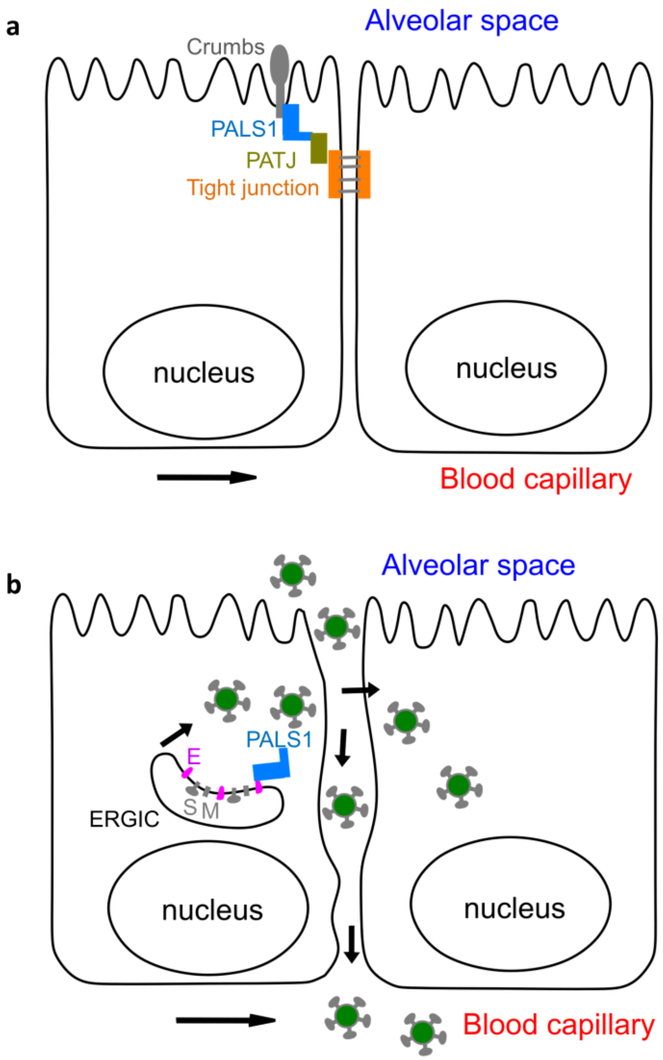

Supplementary Fig. 1. A model of E-mediated PALS1 relocalization and vascular damage. **a** A schematic drawing of two adjacent lung epithelial cells with the Crumbs apical complex maintaining cell polarity and tight junction formation. **b** SARS-CoV-2 E protein interacts with PASL1 and recruits it to the ERGIC site, causing vascular leakage and damage to cell junctions, promoting viral spread and cytokine storm, and leading to ARDS and in some cases deaths.

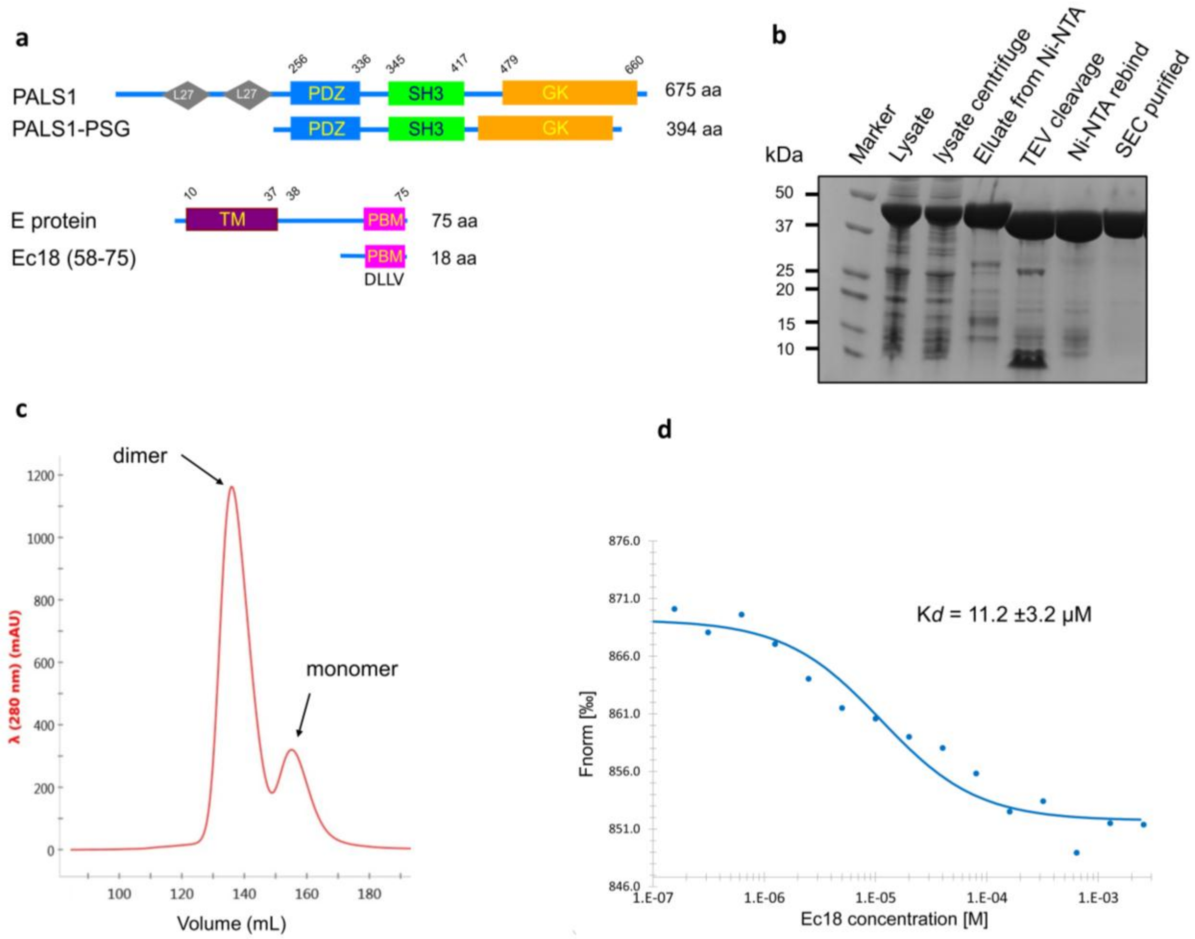

Supplementary Fig. 2. Protein production. a Schematics of the domains in PALS1 and E. PALS1-PSG and Ec18 were used in this work. **b** SDS-PAGE analysis for the purification of PSG. Source data are provided as a Source Data file. **c** SEC analysis for the purification of the PSG dimer. **d** Interactions between PALS1-PSG and Ec18 measured by microscale thermophoresis (MST). Fnorm is a normalized value which relates to fluorescence values before and after laser activation. PALS1-PSG was labeled by a fluorescence dye NT-647 and was titrated using Ec18 at different concentrations. The fitted K*d* is 11.2 ±3.2 μM. The experiment was repeated twice with similar results from different samples.

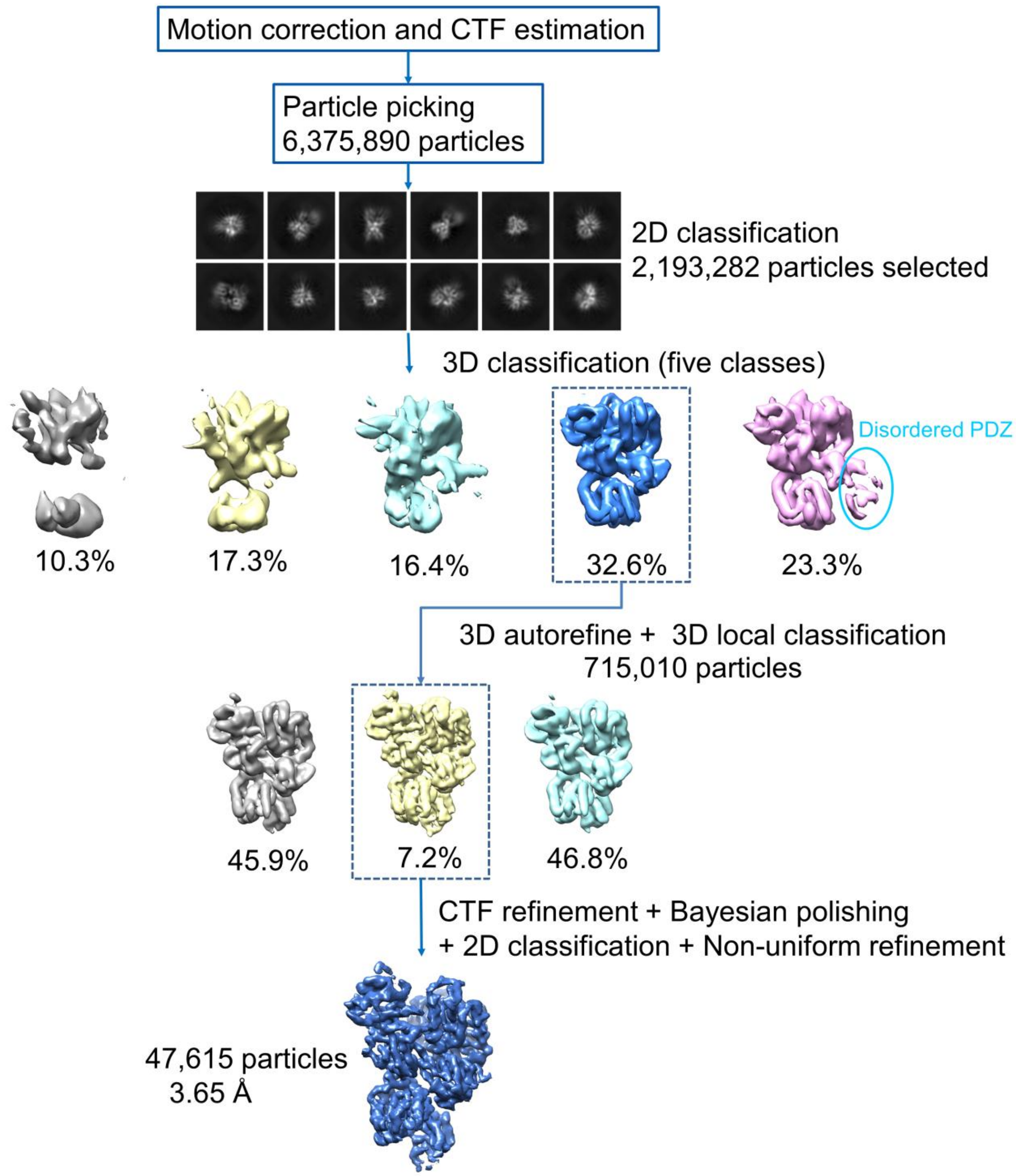

Supplementary Fig. 3. A data analysis workflow.

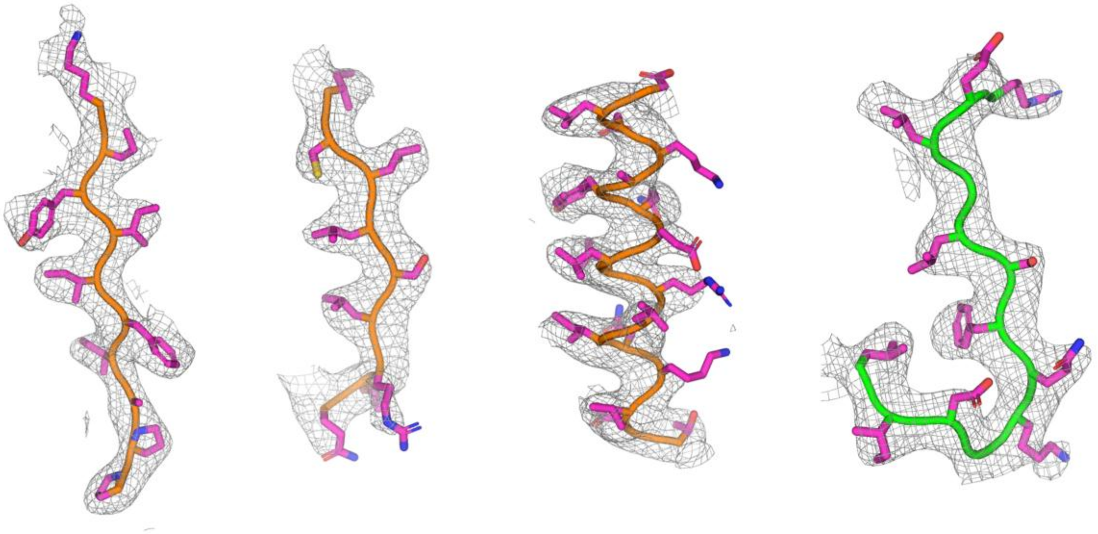

Supplementary Fig. 4. Quality of densities showing secondary structures and side chains.

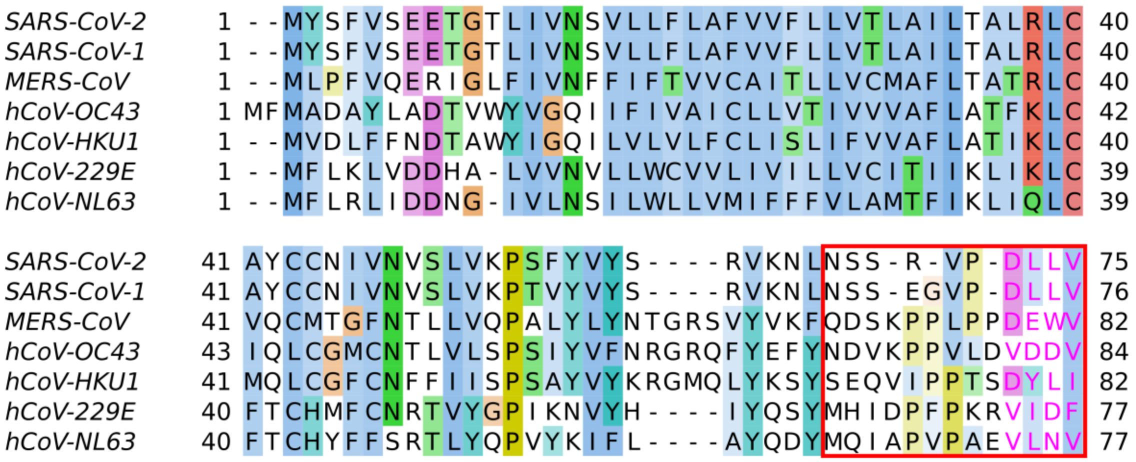

Supplementary Fig. 5. Multiple sequence alignment of seven human coronavirus E proteins. The sequences of human coronavirus (hCoV) E proteins were downloaded from UNIPROT (www.uniprot.org) with access entry names P0DTC4 for SARS-CoV-2, P59637 for SARS-CoV-1, K9N5R3 for MERS-CoV, Q04854 for hCoV-OC43, Q5MQC8 for hCoV-HKU1, P19741 for hCoV-229E, and Q6Q1S0 for hCoV-NL63. C-terminal PBM sequences were colored in magenta. Red box indicates the C-terminal segments with large sequence variations among the seven hCoVs. The multiple sequence alignment is prepared in Jalview (www.jalview.org).

Supplementary Table 1. Cryo-EM data collection, 3D reconstruction, and refinement statistics.

**Data Collection**	
Microscope	Titan Krios G3i
Stage type	Autoloader
Voltage (kV)	300
Detector	Gatan K3
Energy filter (eV)	20
Acquisition mode	Super-resolution
Physical pixel size (Å)	0.684
Defocus range (µm)	0.7-2.5
Electron exposure (e^-/Å^2)	64
**Reconstruction**	
Software	Relion v3.08, CryoSPARC v2.15
Particles picked	6,375,890
Particles final	47,615
Extraction box size (pixels)	256
Rescaled box size (pixels)	64
Final pixel size	0.684
Map resolution (Å)	3.65
Map sharpending B-factor (Å^2)	100
**model refinement**	
Software	PHENIX
Refinement algorithm	Real Space

Clipped box size (pixels)	None
Number of residues	627
R.m.s deviations	
Bond length (Å)	0.007
Bond angle (°)	0.774
Molprobity clashscore	9.04
Rotamer outliers (%)	0.0
Cβ deviations (%)	0.0
Ramachandran plot	
Favored (%)	85.65
Allowed (%)	14.35
Outliers (%)	0
PDB code	7M4R
