# Supplementary figures and images for: Structural basis for SARS-CoV-2 envelope protein recognition of human cell junction protein PALS1
Source: Nat Commun. 2021 Jun 8;12:3433. doi: 10.1038/s41467-021-23533-x (PMC8187709; doi:10.1038/s41467-021-23533-x)

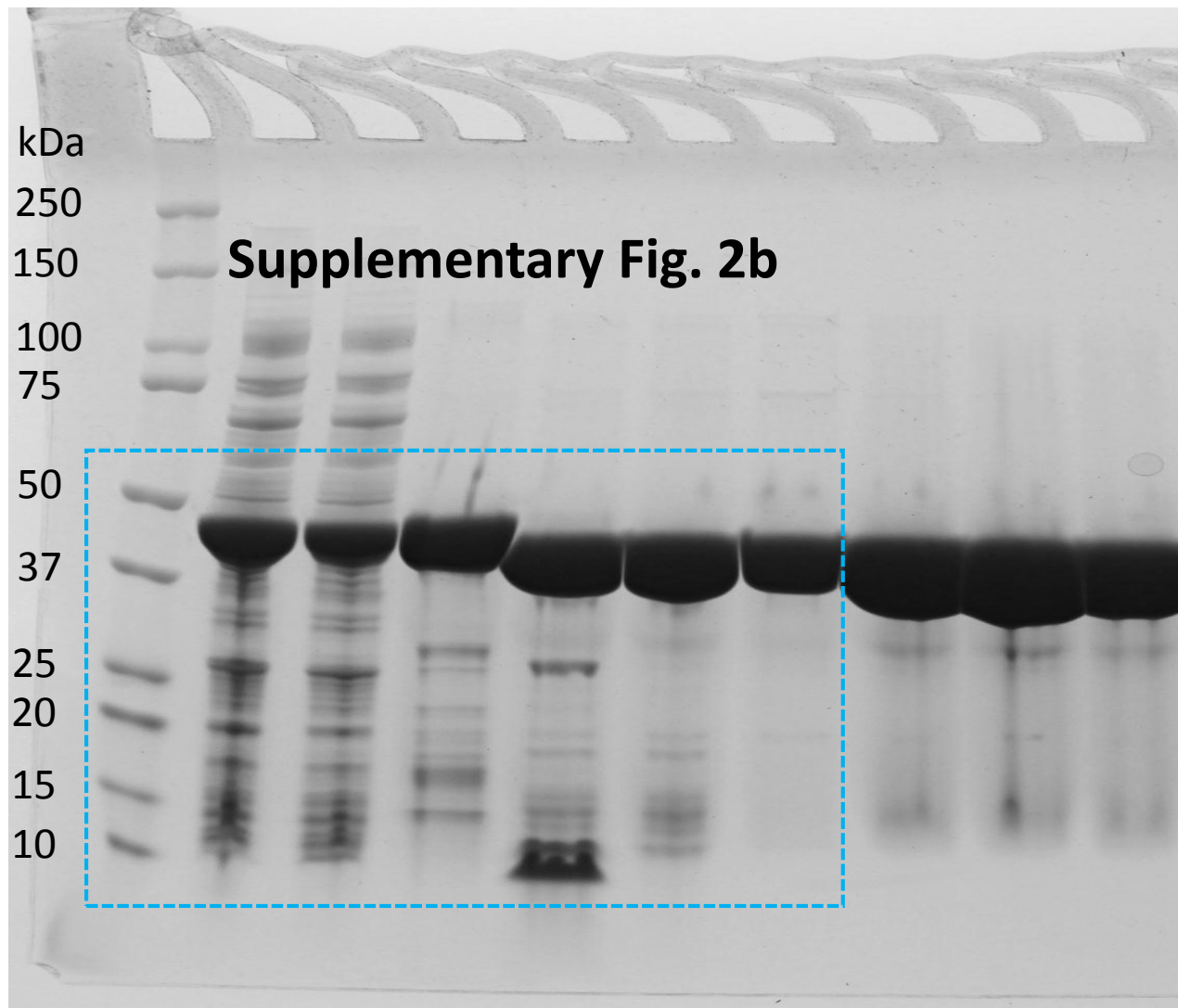

Supplementary Fig. 2b in the manuscript is marked in a dashed rectangle.

Supplement: Supplementary file 4 — Source Data [file 41467_2021_23533_MOESM4_ESM.pdf]
